# Effectiveness of topical vancomycin in the prevention of spinal surgical site infections: a retrospective cohort study

**DOI:** 10.1186/s13756-021-01006-6

**Published:** 2021-09-26

**Authors:** Rawan T. Tafish, Ahmed F. Alkhaldi, Anouar Bourghli, Turki A. Althunian

**Affiliations:** 1 Kingdom Hospital and Consulting Clinics, Riyadh, Saudi Arabia; 2 Saudi Food and Drug Authority, Riyadh, Saudi Arabia

**Keywords:** Topical vancomycin, Spinal surgery, Surgical site infections, Antimicrobial resistance, Cohort study, Effectiveness

## Abstract

**Background:**

The risk of surgical site infections (SSIs), particularly methicillin-resistant *Staphylococcus aureus* (MRSA) SSIs, after spinal surgeries is one of the most daunting experiences to patients and surgeons. Some authors suggest applying vancomycin powder on the wound before skin closure to minimize the risk of SSIs; however, this practice is not supported by well-established evidence. This study sought to assess the effectiveness of topical (i.e. intra-wound) vancomycin in minimizing the risk of SSIs in patients who underwent spinal surgeries at a Saudi hospital.

**Methods:**

A retrospective cohort study was conducted using the hospital database. Patients who underwent spinal surgeries from the period of 09/2013 to 09/2019 were included and followed up (observed from the time of the surgery) to 30 days (surgeries without implants) or 90 days (with implants). The odds ratio (OR) of the primary outcome between vancomycin treated versus non-treated patients was estimated using a logistic regression model adjusting for the measured confounders. A sensitivity analysis was conducted using propensity score analysis (inverse probability of treatment weighting [IPTW] with stabilized weights) to control for confounding by indication. All study analyses were completed using RStudio Version 1.2.5033.

**Results:**

We included 81 vancomycin treated vs. 375 untreated patients with 28 infections (8/81 vs. 20/375; respectively). The adjusted OR of SSIs between the two groups was 0.40 (95% confidence interval [CI] 0.11 to 1.34). The result of the propensity score analysis was consistent (OR: 0.97 [95% CI 0.35 to 2.68]).

**Conclusions:**

We could not find a lower association of SSIs with intra-wound vancomycin in patients who underwent spinal surgeries. Further studies are needed to assess benefits of using topical vancomycin for this indication vs. the risk of antimicrobial resistance.

## Background

Surgical site infections (SSIs) are the third most common complication among patients who underwent spinal surgeries with an overall prevalence of 3.1% (the prevalence is up 13.0% in the highest risk group) [1–3]. SSIs are defined as infections occurring after 30 days from the operative procedure or up to 90 days in complicated deep incisions requiring implants [4]. They are burdensome complications and associated with a high risk of morbidity (especially readmission), mortality and economic loss [5]. In the United States, the direct and indirect health cost of SSIs after spinal surgeries is up to 10 billion dollars with a mortality rate of 8,000 deaths per year [6]. The risk is the highest among patients with thoracic spinal procedures (3.7%), followed by the cervical and lumbar procedures (3.4 and 2.7%), respectively [1]. The benefits of some preventive measures against SSIs using prophylactic antibiotic regimens (with cefazolin as a fixed component) have been outweighed by the risk of methicillin-resistant *Staphylococcus aureus* (MRSA) and coagulase-negative staphylococci (CoNS) [7–13]. This led to the off-label use of topical vancomycin (i.e. intra-wound) in some settings [14–44]

The off-label topical application of 1–2 g vancomycin powder from the injectable dosage forms for the prevention of SSIs after spinal surgeries is controversial [14–44]. The published studies on the effect of this application in the prevention of SSIs after spinal surgeries have shown mixed results, and none was conducted in Saudi Arabia (a recent study showed that the prevalence of MRSA infections was 19.1% at a Saudi tertiary hospital) [14–45]. Additionally, the design of these studies was subjected to several methodological limitations (e.g. the absence of a control group, the suboptimal choice of the control group, the suboptimal definition of the outcome, the suboptimal adjustment of potential measured confounders, ignoring the impact of some known effect modifiers, etc.). The aim of this study was to assess the effectiveness of topical vancomycin in the prevention of SSIs after spinal surgeries taking into consideration the aforementioned design limitations in a Saudi population.

## Methods

### Source of data

This retrospective cohort study was conducted using the database of a private hospital (the Kingdom Hospital) in Riyadh, Saudi Arabia. Data from the routine clinical care are added to the hospital’s electronic health records. Diseases are coded using the International Statistical Classification of Diseases and Related Health Problems 10th Revision (ICD-10). Patient socio-demographic details, surgery type, relevant lab investigations (including culture sensitivity results), re-admission, follow-up periods and patient outcome were collected through a direct access to the electronic health records. Original patient medical charts were also accessed to collect missing values of the aforementioned variables and to collect data about additional covariates (past medical history, operation time, topical drug/intra-operative systemic drug exposure, and vital signs).

### Study cohort

Patients who underwent spinal surgeries from the period of 09-2013 to 09-2019 were included. All patient visits to the hospital during the 90-day period after spinal surgeries were observed (either as scheduled or non-scheduled visits). Any infection (signs, symptoms and documented positive bacterial culture findings) that occurred within 30 days after the surgery (patients without implants) or 90 days (patients with implants) was considered as an SSI after spinal surgery (i.e. the outcome of the study). The infection was classified as deep or superficial based on the definition of the United States Center for Disease Control and Prevention (CDC) [46]. According to the CDC, superficial SSIs involve only the skin layer; other more serious SSIs which involve tissues under the skin, organs or implanted material can be considered as deep [46]. This follow-up period is reflective of the maximum follow-up period stated in the definition of SSIs after spinal surgeries in the “Introduction” section. All types of spinal surgeries were included in the study (i.e. lumbar, thoracic, thoracolumbar, and cervical) using the anterior or the posterior approach.

### Study exposure groups and outcome

The included patients in this study were split into two exposure groups: a group with incident topical vancomycin treatment (test group) and another group of patients who were not treated with topical vancomycin (control group). The cohort entry date was defined as the date of the spinal surgery. The outcome was the first SSI observed in the follow-up period. For all patients in the test group, two-third of the vancomycin powder was first spread directly on the bones and muscles at the end of the surgery, then the remaining amount was applied between the fascia and the fat layer after fascia closure. Doses of vancomycin varied between 0.5 and 2 g based on the wound size and the type of the surgery, but the majority of patients received 1 g (61 of 81 [75%]). MRSA screening was performed for 19 patients before the surgery, and colonization was identified in two patients (one received topical vancomycin). None of these two patients received any treatments before the surgery.

### Confounders

The analysis of the study outcome was adjusted for the following potentially confounding variables: age, sex, body mass index (BMI), type of spinal surgery, smoking status, diabetes mellitus, kidney functions, hypertension, history of spinal surgeries, history of antibiotic use within 90 days before cohort entry date, prolonged operation time (an operation lasting 100 min or more), surgical approach (anterior vs. posterior), implants, use of disinfectants (alcohol, povidone-iodine, chlorhexidine), topical gentamicin solution for irrigation of wounds (i.e. applying the solution over the operation site) as one of the SSI prophylactic measures, pre- and post- operative inflammatory/infection markers such as white blood cell counts and neutrophil counts. These potential confounders were reported in the literature as potential risks for the outcome of the study with an assumption that none of them is an instrumental variable [1, 47–59].

No prolonged antibiotic treatment was given to any of the patients in this study (as recommended by the hospital guideline). All patients were given a prophylactic systemic cefazolin intravenous injection within 60 min before the scheduled surgeries (doses of 1–2 g with patients above 80 kg receiving the 2 g dose). The systemic prophylactic cefazolin dose was repeated in case of high blood loss or during a long surgical intervention (i.e. if the surgery time exceeded two cefazolin half-lives). All surgeries in the study were performed by the same surgeon.

### Statistical analysis

The primary hypothesis of the study was that topical vancomycin use is superior to non-vancomycin use in the prevention of SSIs after spinal surgeries. This hypothesis was tested using a logistic regression model adjusting for the measured confounders. The observed number of the primary outcome events was low; therefore, the odds ratio (OR) estimated from the model would provide a close approximation of the relative risk.

During the study period, there was a tendency to use intraoperative topical vancomycin for patients who are at high risk of SSIs after spinal surgeries (those who were scheduled for implants or those with a history of spinal infections). Thus, a sensitivity analysis was conducted using propensity score analysis to take into account the possible risk of confounding by indication [60–63]. In the first step, propensity scores (the probability of assignment to vancomycin group) for the included patients were estimated using a logistic regression model incorporating all study measured confounders. Then, a propensity score analysis was conducted using inverse probability of treatment weighting (IPTW) with stabilized weights. The balance check in the new “pseudo-population” was based on the absolute standardized difference (a balance was achieved if the difference was < 10%). All statistical analyses were conducted using RStudio Version 1.2.5033.

## Results

We included 456 patients who were either treated with topical vancomycin (n = 81) or without an exposure to topical vancomycin (n = 375) (Table 1. Most patients underwent lumbar surgeries (343 of 456 [75%]), most surgeries had a posterior approach (425 of 456 [93%]), and more than two-third of patients were given prophylactic gentamicin solution for irrigation (349 of 456 [77%]). None of the patients experienced systemic adverse events.

**Table 1:** Baseline characteristics of the study groups

Baseline characteristics	Vancomycin treated N = 81	Vancomycin untreated N = 375	Difference (*p*-value)
*Age (years)*			
Median	50	45	0.06
IQR	27	22	
*Sex no. (%)*			
Male	47 (58.0)	249 (66.4)	0.19
Female	34 (42.0)	126 (33.6)	
*BMI (kg/m*^*2*^*)*			
Median	29.7	29.4	0.52
IQR	8.4	7.2	
*Type of surgery no. (%)*			
Lumbar	58 (72.0)	285 (76.0)	< 0.01
Thoracic	5 (6.0)	5 (1.0)	
Thoracolumbar	15 (18.0)	30 (8.0)	
Cervical	3 (4.0)	55 (15.0)	
*Smoking history no. (%)*			
Never smokers	60 (74.0)	232 (62.0)	0.12
Current smokers	19 (23.0)	125 (33.3)	
Former smokers	2 (3.0)	18 (5.0)	
Diabetes mellitus no. (%)	20 (25.0)	64 (17.0)	0.14
*Renal functions no. (%)*			
Normal	79 (98.0)	371 (99.0)	0.28
Abnormal	2 (2.0)	4 (1.0)	
Hypertension no. (%)	29 (36.0)	98 (26.0)	0.10
History of spinal infection no. (%)	4 (5.0)	3 (1.0)	0.02
History of spinal surgeries no. (%)	19 (23.0)	54 (14.0)	0.06
History of antibiotic use within 90 days before index date no. (%)	13 (16.0)	28 (8.0)	0.03
Prolonged operation time no. (%)	71 (88.0)	29 (8.0)	< 0.01
*Surgical approach no. (%)*			
Anterior	0 (0.0)	31 (8.0)	< 0.01
Posterior	81 (100.0)	344 (92.0)	
Implant no. (%)	72 (89.0)	184 (49.0)	< 0.01
Alcohol as disinfectant no. (%)	70 (86.0)	303 (81.0)	0.30
Prophylactic gentamicin solution for irrigation no. (%)	68 (84.0)	281 (75.0)	0.11
*Pre-operative white blood cell counts (in 10^3/µl)*			
Median	7.5	8.2	0.08
IQR	3.8	3.1	
*Pre-operative neutrophil counts (%)*			
Median	54.7	57.4	0.13
IQR	13.9	13.9	

**Fig. 1:**
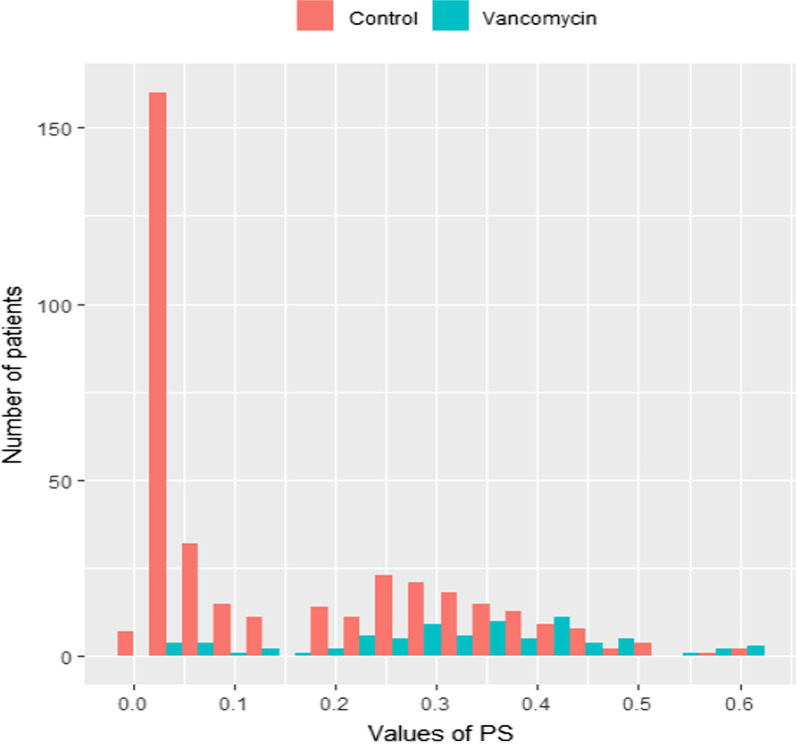
Propensity score distribution in the vancomycin (test) vs. control (non-user) groups

A total of 28 cases of SSIs after spinal surgeries were identified in vancomycin treated vs. untreated groups (8 of 81 [9.9%] vs. 20 of 375 [5.3%], respectively), and the median time to the occurrence of the infection was 14 days. Out of 28 infections, 16 (57%) were considered deep and 12 (43%) were superficial (Table 2). Some patients were infected by more than 1 isolated pathogens which explains the higher number of the total isolated microbes compared with the number of SSI cases (36 vs. 28 respectively), (Table 3). The microbiological analysis revealed that 50% (14/28) of these SSIs were caused by *Staphylococcus* species (4 vs. 10 in vancomycin treated and untreated groups; respectively), (Table 3). The other 50% were caused mostly by Gram negative bacteria such as *Klebsiella pneumoniae*, *Pseudomonas aeruginosa*, and *Escherichia coli* isolates (Table 3). The distribution of Gram positive and polymicrobial/Gram negative SSIs was similar between vancomycin treated vs. untreated groups. The odds of SSIs in the vancomycin group was comparable to the odds in the non-exposure group: OR = 0.40 (95% confidence interval [CI] 0.11 to 1.34). The results of propensity score estimation showed a clear evidence of confounding by indication (i.e. a large proportion of the patients in the non-exposure group had a propensity of zero) as shown in Fig. 1, which also prevented trimming of the extreme values. The balance was achieved in 50% of the variables in the pseudo-population. The result of the IPTW analysis was consistent with the results of the first analysis; however, the direction of the point estimate shifted to the null value: OR = 0.97 (95 %CI 0.35 to 2.68).

**Table 2:** Microbiological analysis of the identified SSIs

Isolated microbe	Deep infections (n)	Superficial infections (n)
*Mycobacterium tuberculosis*	2	0
Methicillin susceptible *Staphylococcus aureus*	3	5
*Klebsiella pneumoniae*	1	0
*Klebsiella pneumoniae* –ESBL^*^	2	1
*Pseudomonas aeruginosa*	4	2
*Escherichia coli*	0	2
*Enterobacter cloacae*-MDR**,	1	0
*Morganella morganii*	2	0
*Acinetobacter baumannii*	1	0
MRSA	4	0
*Escherichia coli* -ESBL*	1	1
Coagulase-Negative *Staphylococci*^±^	0	4
Total number	21	15

*ESBL extended-spectrum beta-lactamase

**MDR multi-drug resistant

±Coagulase negative staphylococci: two isolates were Staphylococcus epidermidis and Staphylococcus hominis. The other two isolates were not identified

**Table 3:** Isolated microbes in infected Vancomycin treated and Vancomycin untreated patients

Isolated microbe*	Total number of isolates N = 36	Vancomycin treated N = 11	Vancomycin untreated N = 25
Methicillin-susceptible *Staphylococcus aureus* no. (%)	8	1 (9%)	7 (28%)
MRSA no. (%)	4	2 (18%)	2 (8%)
Coagulase-negative *Staphylococci* no. (%)^±^	4	2 (18%)	2 (8%)
*Klebsiella pneumoniae*	4	1 (9%)	3 (12%)
*Escherichia coli*	4	2 (18%)	2 (8%)
*Enterobacter cloacae*	1	0 (0%)	1 (4%)
*Morganella morganii*	2	1 (9%)	1 (4%)
*Pseudomonas aeruginosa*	6	1 (9%)	5 (20%)
*Mycobacterium tuberculosis*^&^	2	0 (0%)	2 (8%)
*Acinetobacter baumannii*	1	1 (9%)	0 (0%)

*Samples for culture sensitivity were taken from a swab as deep as possible during the follow-up visits upon the presence of signs/symptoms of infection. Tissue samples (e.g. bone or fascia) were taken in the operation room under general anesthesia in case of revision/debridement. Species confirmation was performed using Vitek® System

±One isolate was methicillin- susceptible Staphylococcus hominis, the other was methicillin- resistant Staphylococcus epidermidis. The species of the other two isolates were not identified, but one was methicillin resistant and the other was methicillin susceptible

^&^Assessment for Mycobacterium tuberculosis was performed for patients with signs/symptoms and/or history of tuberculosis infection

## Discussion

This study found a comparable risk of SSIs after spinal surgeries between the topically vancomycin treated vs. untreated patients. Nevertheless, the findings of the original and sensitivity analyses showed that the usefulness of topical vancomycin in this context would benefit from the conduct of additional multicenter studies with the inclusion of a larger number of patients.

The risk of SSIs after spinal surgeries between the vancomycin treated vs. untreated groups in our study was found to be comparable, which was similar to the findings of two-third of the published studies (20 of 30 [67%]) that assessed the effectiveness of vancomycin in this patient group [14–44]. The risk in the topical vancomycin group in the largest of those studies varies from being lower, to comparable, or even to higher vs. the groups who were not treated with topical vancomycin. Nevertheless, the analysis of this risk in most of the published studies was not adjusted for the known measured confounders, and the risk of confounding by indication was taken into account only in two of these studies [25, 27, 28, 32, 38]. Additionally, the risk in two of the largest studies was assessed without a control group (i.e. in a pre and post fashion) [25, 27]. The evidence from three randomized controlled trials was not conclusive since two trials were open-label, and two of were stopped prematurely [17, 37, 41]. The risk of SSIs in our study was comparable to that in the two studies in which confounding by indication was taken into account by propensity score analysis [28, 38]. The propensity score model in our study showed that the use of topical vancomycin is being channeled to the group of patients who are at higher risk of SSIs after spinal surgeries. The effect of confounding by indication was also clear in the shift of the effect from an OR of 0.4 in the logistic regression towards the null value (OR = 0.97) in the IPTW analysis.

Doses of topical vancomycin in the published studies ranged from 0.5 to 2 g, most utilized a prophylactic cefazolin dose, and most were designed as retrospective cohort studies. It is anticipated that the effect of the topical vancomycin would be instantaneous and would last for the entire follow-up period (the concentrations are known to be high with a low systemic absorption) [64]. The findings of our study showed that the distribution of Gram positive and polymicrobial/Gram negative SSIs was similar in both groups. This was different from their distribution in most published studies in which the risks of Gram positive pathogens (i.e. *Staphylococcus* species) and polymicrobial/Gram negative pathogens in the vancomycin-treated group were lower and higher, respectively [42, 43]. No systemic adverse events were observed in our study which is consistent with the findings from the literature (only two of over 2000 topical vancomycin administrations were associated with systemic adverse events) [64].

Our study was the first to assess the effectiveness of topical vancomycin in minimizing the risk of SSIs after spinal surgeries in the Gulf region. Additionally, it was one of the few observational studies in this context that took confounding by indication into account with a good level of completeness of the measured confounders. Another strength in the study was the sufficient follow up period (30–90 days) giving that 30-days follow up by the same surgeon was completed for almost 94% of the patient population, rendering the risk of outcome misclassification. The main limitation in our study was that it was limited to a single center. Addtionally, the lack of randomization prevented us from having a structured intervention and documentation (as well as adjusting for all types of confounders). The inclusion of more centers (especially the largest ones) in future studies would improve the generalizability of the assessment.

## Conclusions

To conclude, we could not find a lower risk of SSIs after spinal surgeries with the use of topical vancomycin. Further studies are needed to assess benefits of using topical vancomycin for this indication vs. the risk of antimicrobial resistance.

## Data Availability

The datasets generated and/or analysed during the current study are not publicly available due [patient confidential data] but the anonymized datasets are available from the corresponding author on reasonable request.

## Abbreviations

SSIs: Aurgical site infections
MRSA: Methicillin-resistant *Staphylococcus aureus*
OR: Odds ratio
IPTW: Inverse probability of treatment weighting
ICD-10: International Statistical Classification of Diseases and Related Health Problems 10th Revision
BMI: Body mass index
